# Electrochemotherapy as a symptom-oriented palliative treatment option: an exploratory single-center study in 15 patients with locally advanced or locoregionally recurrent vulvar carcinoma

**DOI:** 10.1007/s00404-025-08296-w

**Published:** 2026-01-14

**Authors:** Eva-Maria Grischke, Felix Neis, Ernst Oberlechner, Melanie Henes, Martin Weiß, Sara Y. Brucker

**Affiliations:** Department of Obstetrics and Gynecology, University of Tübingen, Calwerstr. 7, 72076 Tübingen, Germany

**Keywords:** Oncology, Chemotherapy, Female genital tract, Female external genitalia

## Abstract

**Background:**

Vulvar carcinoma (VC), a rare cancer, recurs in over a third of women, usually within 2 years. Treatment options other than repeat surgery and radiotherapy are often required in the recurrence setting. Systemic chemotherapy is an option but is generally associated with stressful side effects. In the palliative setting, electrochemotherapy (ECT) is a better-tolerated alternative, which provides local tumor control while obviating systemic side effects.

**Objective:**

To descriptively analyze a case series of patients with inoperable locally advanced or locoregionally recurrent VC receiving bleomycin-based ECT.

**Methods:**

Descriptive analysis of prospectively collected data from a case series. Postmenopausal women with locally advanced or locoregionally recurrent VC were eligible for inclusion. Bleomycin was administered at 15 mg/m^2^ body surface as a single 1-min intravenous injection; 8 min later, electrochemical treatment using sterile disposable electrodes was performed under anesthesia for ≤ 30 min. Postoperatively, patients received pain medication to mitigate muscle soreness.

**Results:**

15 patients were included in the study. Median patient age at ECT was 81 (range, 51–100) years. Recurrences (1–5) were present in 12 patients. Surgery and radiotherapy were not justifiable options in 3 patients. In our clinical observation, ECT was well tolerated by all patients for the management of pain, itching, odor, and secretion. This allowed for time to be gained until further treatment became necessary or disease progression occurred.

**Conclusions:**

In our clinical experience, bleomycin-based ECT is an oncologically efficacious and better-tolerated alternative to systemic chemotherapy or immunotherapy in patients with recurrent VC in a palliative setting with limited capacity to undergo treatment.

## What does this study add to the clinical work

**Table:** 

This study has demonstrated that electrochemotherapy represents an important expansion of the therapeutic spectrum in the treatment of vulvar carcinoma. Particularly in older patients with numerous comorbidities, a significant reduction in distressing symptoms such as discharge, pain, and itching can be achieved, both as part of primary treatment and as an adjunct therapy.	

## Introduction

Accounting for about 5% of malignant gynecological neoplasms, vulvar carcinoma is a rare tumor entity, which however, represents a significant clinical problem particularly in elderly patients aged > 75 years [1].

Recurrences of vulvar carcinoma occur locally in vulva, lower vagina, and regional lymph nodes, or as distant metastases. Recurrences develop in over a third of women, most frequently within 2 years. Even in stage 1A, local recurrence rates of 5% and more have been reported [2]. More than half of recurrences occur as isolated lesions of the vulva/perineum, whereas inguinal femoral lymph nodes or pelvic lymph nodes are less frequently affected. Patients with a local recurrence have a considerable risk of developing a subsequent local recurrence; less than 10% of recurrences are distant metastases [3–7].

Depending on the primary treatment received and the extent of the recurrence, the aim is generally to remove the lesions locally along with a margin of healthy tissue. In the case of larger recurrences or after extensive resection during primary treatment, flap plasty may be necessary for tension-free defect coverage. However, if primary surgery is very extensive, the option of further local surgical treatment is very limited. There are no established recommendations on the tumor-free excision margins for the surgical treatment of local recurrences of vulvar carcinoma. In the case of advanced local recurrences, in which surgical removal of tumor tissue with a healthy margin is unlikely, radio-chemotherapy is usually or frequently indicated. If this therapeutic option has also been exhausted, the next step is to consider exenteration. However, this is not an option for patients of advanced age or those with numerous comorbidities. The possibility of systemic therapy may also be considered but is generally associated with numerous side effects and, therefore, often stressful.

Electrochemotherapy (ECT) is a minimally invasive locoregional therapy that has been shown to be effective in a broad range of cutaneous malignancies of various histotypes, including metastasized breast cancer [8, 9]. For patients with recurrent or locally advanced vulvar carcinoma, local ECT may provide a viable treatment option that can stabilize the disease and achieve local tumor control in terms of pain alleviation and reduction of ulceration, odor formation, and secretion without the side effects of systemic chemotherapy or immunotherapy. To explore the applicability of ECT, our study sought to prospectively collect and analyze data from patients with recurrent or locally advanced vulvar carcinoma treated at our hospital from 06/2014 through 09/2023.

## Methods

### Study design and ethics

This study was a descriptive analysis of prospectively collected treatment data from a series of patients receiving bleomycin-based ECT for inoperable locally advanced or locoregionally recurrent vulvar carcinoma in a palliative setting. The primary objective of the study was to collect data on local tumor control and the control of clinical signs and symptoms, including ulceration, pain, secretion, itching, and odor formation. Index lesions were longitudinally documented by photography. Subsequent evaluation of the images was performed in a blinded manner.

This study was approved by the Ethics Committee of the Medical Faculty of the University of Tübingen and Tübingen University Hospital (approval no. 558/2015BO1), who confirmed that no specific approval was required as this was an observational study. Patients provided their written informed consent to study participation and the use of their nonidentifiable data for research and publication purposes. In patients with moderate-to-severe dementia who were under legal guardianship, consent was obtained from the patients’ legal guardians.

### Patients

Included in the present study were postmenopausal women with histologically confirmed, cutaneously accessible, inoperable, locally advanced or locoregionally recurrent vulvar carcinoma (≤ 5 lesions, 1–5 cm in diameter; thickness ≤ 3 cm) who underwent bleomycin-based ECT as a palliative treatment at our hospital during the period between 06/2014 and 09/2023. Patients who failed to provide written informed consent as a symptom-oriented palliative treatment option were to be excluded from study participation, as were patients with symptomatic or rapidly progressing metastasis outside the region of the vulva. Medications and underlying or concomitant diseases were not considered exclusion criteria.

### Treatment

ECT was performed as a palliative treatment using electroporation and a single 1-min intravenous administration of bleomycin at a dose level of 15 mg per m2 of body surface, in accordance with market authorization and standard operating procedures.

**Fig. 1:**
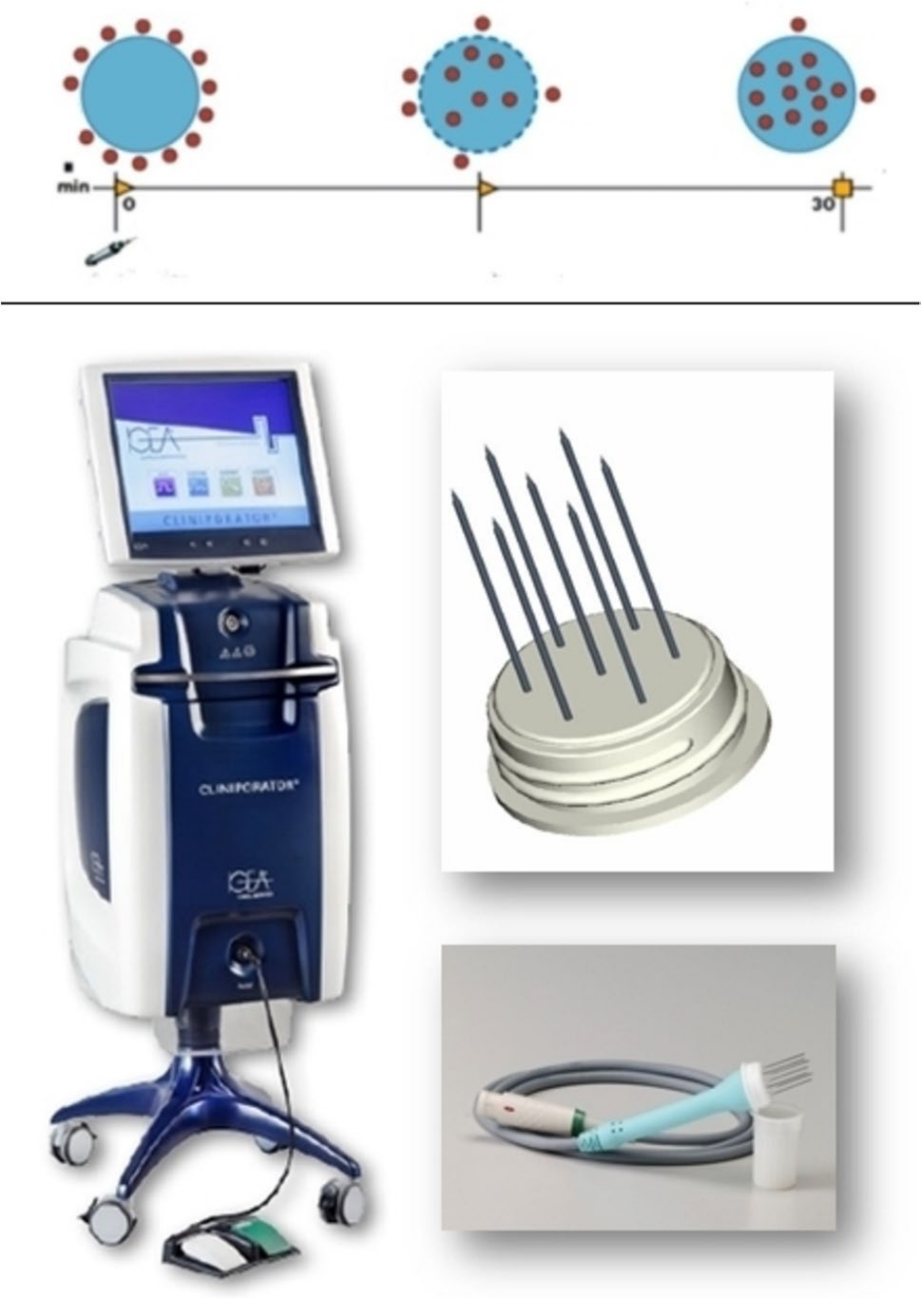
Electrochemotherapy: mode of action and equipment. Mode of action (top, left to right): drug molecules reach a tumor cell; electroporation increases cell membrane permeability, enhancing drug uptake; cell membrane pores close again once the delivery of electric pulses has been completed, thus enabling the drug to exert its cytotoxic action intracellularly. Equipment: CLINIPORATOR® VITAE control unit (bottom left; IGEA, Carpi (MO), Italy) and hexagonally configurated electrode, and handle and cable (bottom right; equipment images by courtesy of the manufacturer)

Local electrochemical treatment using the CE-certified CLINIPORATOR® VITAE (IGEA, Carpi (MO), Italy) fitted with sterile hexagonally configurated disposable electrodes was initiated 8 min after bleomycin administration. ECT mode of action and equipment are illustrated in Fig. 1. Treatments lasted ≤ 30 min in view of the decreasing effectiveness of intravenous administration. Analgesia was achieved by means of short-lasting, appropriately monitored laryngeal mask anesthesia. Care was taken to keep the administration of oxygen to a minimum to prevent potential pulmonary toxicity from bleomycin.

The vulva as a surgical site is generally contaminated with microorganisms. Therefore, patients received antibiotics in the form of a single injection of a cephalosporin, in combination with an additional intravenous administration of metronidazole in the case of extensive ulcerating lesions. Postoperatively, patients applied sulfadiazine-silver-containing Flammazine® cream (Alliance Pharmaceuticals, Dublin, Ireland) to the treatment site as a local antiseptic.

### Side effect management and subjective tolerability

Postoperatively, the patients also received standard pain medication (level 2 concept) in the form of a nonsteroidal anti-inflammatory drug combined with an opioid, in particular to avoid symptoms such as muscle soreness caused by unpleasant muscle fasciculations during the operation.

### Response assessment and follow-up

Following ECT, patients were monitored for at least 24 h. The main parameters for postoperative follow-up were pain, itching, odor, and secretion. These parameters were recorded in the patients’ case files and subsequent follow-up documentation. Corresponding imaging documentation was also carried out.

### Data collection and analysis

Data collection was based on patients’ medical records and interview-style questioning with regard to pain and quality of life in terms of general wellbeing. Data analysis was of a descriptive nature, based on age at initial diagnosis, age at recurrence, age at primary or recurrence-related ECTs, as applicable, and follow-up data.

## Results

### Patient characteristics

Table 1 summarizes the clinical characteristics, and treatment and follow-up details for all 15 patients.

**Table 1:** Clinical characteristics of 15 ECT-treated patients with vulvar carcinoma

Initial diagnosis (primary tumor) or recurrence no	Age at initial diagnosis, years	Age at ECT, years	Initial diagnosis to 1st ECT	Time from initial diagnosis to 1st ECT, months	Pre-ECT treatment	Post-ECT treatment and follow-up	Recurrence location
0 (primary)	85	85	10/2021–10/2021	0	None	BSC	Vulva, anterior commissure, left labium
0 (primary)	84	84	01/2014–06/2014	6	None	BSC, s/p endometrial carcinoma and uterine sarcoma treated with RTx	Vulva, lower third of the vagina
0 (primary)	92	92	11/2020–06/2021	8	None	BSC	Vulva
1	99	100	10/2019–07/2020	10	RTx	LTFU 2 months after ECT	Vulva
1	84	85	09/2014–06/2015	10	S	LTFU	Left and right groin, introitus, right labium, perineum
1	82	83	12/2019–05/2020	6	RTx	BSC; LTFU	Vulva + mons pubis
1	50	51	03/2018–12/2018	10	RCTx	CTx; PE in 04/2019, deceased in 04/2019	Mons pubis, introitus
2	70	74	04/2011–02/2015	47	S + RTx	BSC	Vulva, left labium
2	69	75	11/2013–01/2020	75	S + RTx	LTFU 2 months after ECT	Mons pubis, labia
2	73	75	09/2021–09/2023	25	S + RTx	Disease progression under immunotherapy with cemiplimab	Left groin
2	79	81	03/2021–03/2023	25	RCTx	No local lesions; nodal disease progression	Mons pubis, transition to the labia
3	63	69	02/2014–05/2019	64	S + RTx	Anterior + posterior exenteration in 11/2020	Vulva
3	67	74	02/2011–10/2017	81	S + RTx	Anterior + posterior exenteration in 04/2018; BSC from 06/2018	Vulva, urethra
3	79	84	02/2011–02/2016	61	S + RTx	CTx until 09/2016; BSC from 01/2017; subsequently LTFU	Mons pubis
5	47	80	02/1983–03/2016	398	S + RTx	CTx	Vulva

*BSC* best supportive care, *CTx* chemotherapy, *ECT* electrochemotherapy, *LTFU* lost to follow-up, *PE* pulmonary embolism, *s/p* status post, *S* surgery, *RCTx* radiochemotherapy, *RTx* radiotherapy

In total, 15 postmenopausal patients with inoperable primary or locoregionally recurrent vulvar carcinoma met the inclusion criteria and underwent ECT during the study period from 06/2014 to 09/2023.

In 3/15 (20%) patients, ECT was the first treatment they received, whereas 12/15 (80%) patients had already developed recurrences, with 8/12 (67%) having experienced their first and second recurrences, 3/12 (25%) having had their third recurrence, and 1/12 (8%) having progressed to the fifth recurrence.

Median patient age at the time of the first ECT was 81 years (range, 51–100 years; interquartile range, 75–85 years; mean age, 79,47 years; standard deviation, 11,07 years). Previous treatments included surgery in 8/12 (75%) patients as the sole primary therapy or primary treatment directly followed by radiotherapy, or radiotherapy as part of the treatment for further recurrences, but these treatments were received before ECT in all instances. 4/12 (33%) patients had undergone radiation therapy alone or in the form of radio-chemotherapy before receiving ECT. In the 3/15 (20%) patients who had not received prior treatment, ECT was the primary treatment option following diagnosis. In 1/15 (7%) of these patients, ECT was performed immediately upon diagnosis, while in the other 2/15 (13%) patients ECT was administered 2 and 5 months after diagnosis (diagnosis-to-ECT 11/2020–06/2021 and 01/2014–06/2014). The decision to perform ECT with the aim of reducing symptoms and stabilizing the disease was made taking into account the patient’s potentially limited ability to undergo more aggressive treatment due to age and comorbidities, and the extension of tumor involvement.

In 4 (33%), 4 (33%), 3 (25%), and 1 (8%) of the remaining 12 patients, ECT was employed to treat the first, second, third, or fifth recurrence, respectively. The earliest recurrence prompting ECT after primary treatment occurred 6 months after the initial diagnosis. The event leading to ECT was the second recurrence in 4/12 patients and occurred up to 25 to 75 months after initial diagnosis. A third recurrence was experienced by 3/12 patients, in these cases up to 61 to 81 months after initial diagnosis of the primary cancer. In 1 (8%) of the 12 patients, the fifth recurrence of the tumor 398 months (33.17 years) after initial diagnosis finally led to ECT.

### Tumor locations

The most common primary location of vulvar carcinoma eventually leading to ECT was the vulva itself, with perineal extension or involvement of the labia also occurring. In 5 patients, the mons pubis was also affected, with extension to the labia or also to the inguinal region. Isolated inguinal involvement was seen in 1 patient with relevant prior treatments in the vulvar region.

### Side effects and subjective tolerability

ECT was observed to be well tolerated. Due to perioperative medication, patients experienced no ECT-induced muscle pain after the intervention.

### Response assessment and follow-up

Considering the patients’ multimorbidity, inpatient stays of up to 2 or 3 days were necessary in some cases. With regard to the individual parameters, i.e., pain, itching, odor, and secretion, the desired therapeutic objective as described above was achieved in all cases.

As regards follow-up treatments after ECT, 2 patients underwent major radical exenteration surgery followed by several additional surgical interventions, taking into account particularly the patient’s age and specific wishes. Systemic treatment, usually chemotherapy, was carried out in 4 patients, in 1 case also targeted immunotherapy. For 10 patients, no further treatment measures were identified in the long-term follow-up (patient loss to follow-up (LTFU)). In the patients with recurrences, the time from initial diagnosis to first ECT ranged between 6 months and over 20 years (80 months), as summarized in Table 1.

In 6 patients, additional treatment consisted of best supportive care. These were the 3 patients in whom ECT was administered as the primary treatment. All three patients, aged 84, 85, and 92, were of a very advanced age. In the other 2 patients, ECT was administered for the first or the second recurrence. These patients were 83 and 74 years old at ECT, respectively. ECT was preceded by radiotherapy and surgery with radiotherapy, respectively.

Four patients subsequently received systemic therapy. In three instances, this involved chemotherapy, and in one case, immunotherapy (with cemiplimab). After ECT, follow-up treatment (in this case, systemic therapy) needed to be started only after 5 or 8 months. In 2 cases, chemotherapy was initiated immediately after ECT, and in 1 case, antibody therapy was started.

### Selected cases

In the case studies cited in the present article, the patients had undergone complex pre-treatment [10, 11]. Recurrences were complex, also affecting regions, such as urethra and perineum. As a rule, these were disseminated lesions.

The following four case histories were selected as examples illustrating the various recurrence sites and medical problems encountered in our study population.

*Patient 1* was a woman aged 75 years at the time of ECT, who had previously undergone vulvectomy with bilateral flap plasty in 2013. She presented with two separate recurrences in the region of the left labia. As shown in Fig. 1 Electrochemotherapy: mode of action and equipment Mode of action (top, left to right): drug molecules reach a tumor cell; electroporation increases cell membrane permeability, enhancing drug uptake; cell membrane pores close again once the delivery of electric pulses has been completed, thus enabling the drug to exert its cytotoxic action intracellularly. Equipment: CLINIPORATOR® VITAE control unit (bottom left; IGEA, Carpi (MO), Italy) and hexagonally configurated electrode, and handle and cable (bottom right; equipment images by courtesy of the manufacturer).

**Fig. 2:**
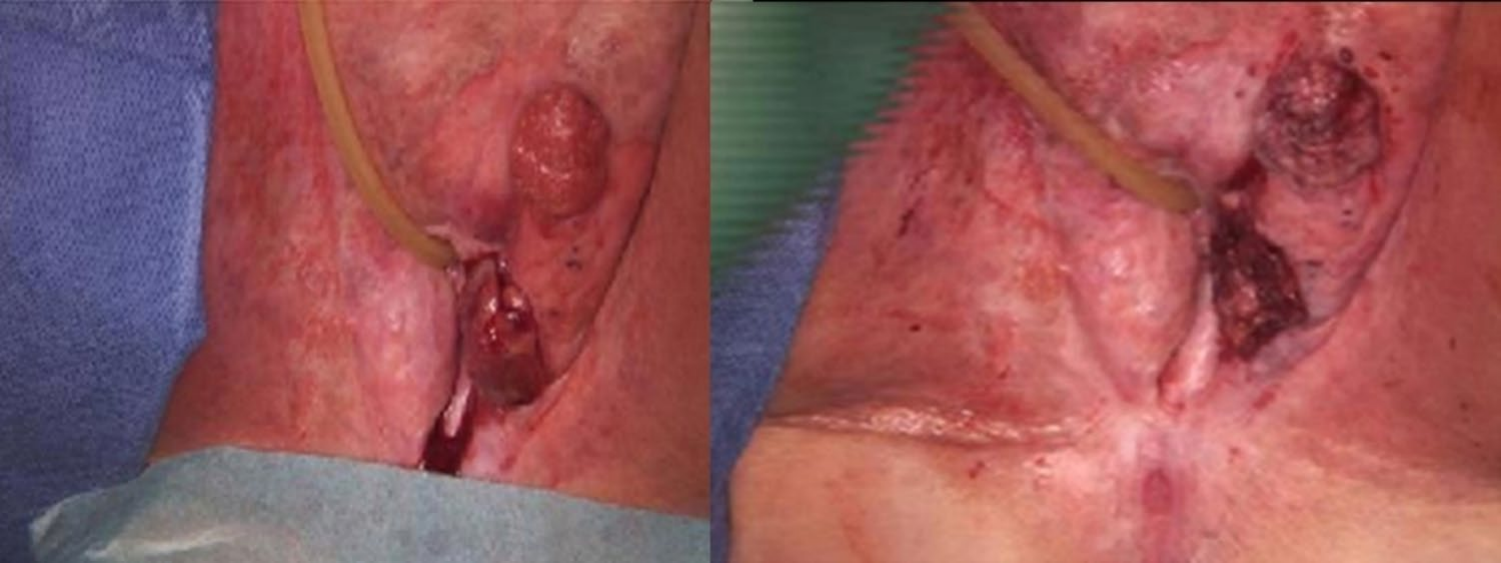
Labial lesions in a 75-year-old patient before and after electrochemotherapy (ECT). The patient had previously undergone vulvectomy with bilateral flap plasty in 2013, 6.25 years before ECT. She presented with two separate recurrences in the region of the left labia. The left and right panels show the operative site before and after ECT, respectively, with the right panel illustrating the extensive ECT-induced destruction of the lesions

Figure 2, the operative site after ECT exhibited extensive destruction of the lesions.

**Fig. 3:**
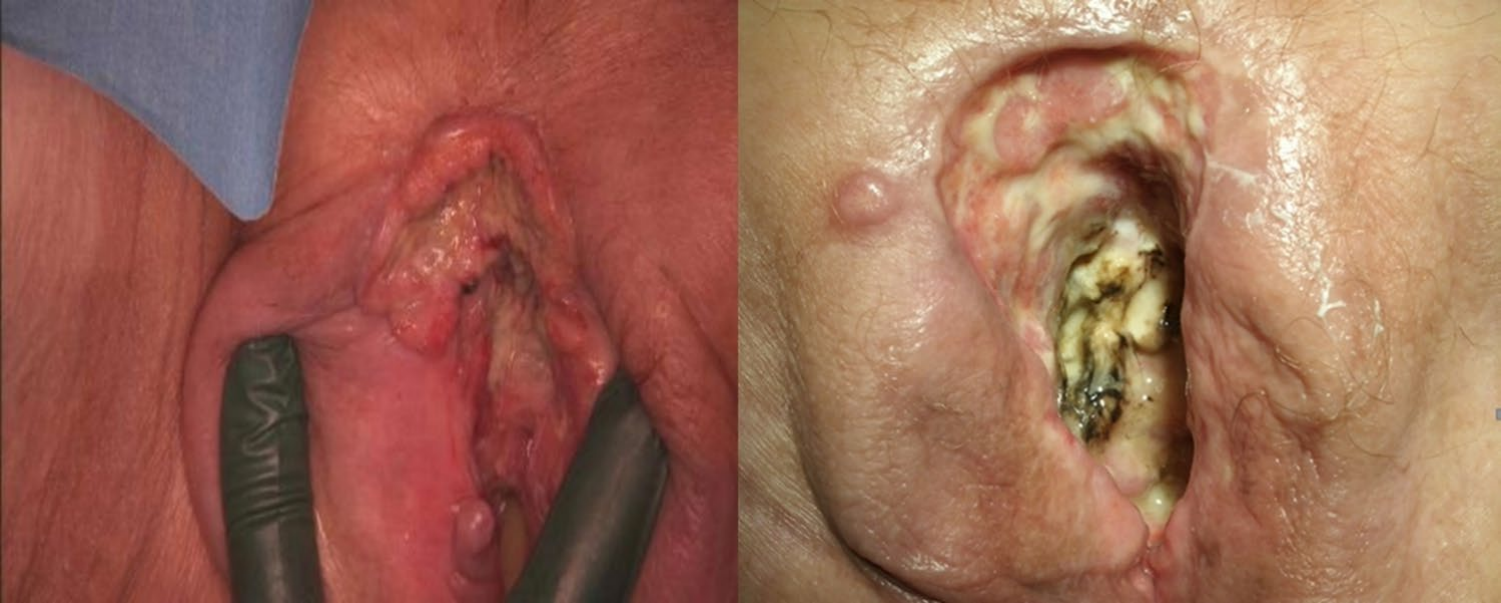
Extensive lesions in the region of the anterior commissure and the introitus before and 3 months after electrochemotherapy (ECT). The patient was a woman with dementia aged 83 years at the time of ECT. She had previously undergone radiochemotherapy as part of her initial treatment upon diagnosis. The extensive lesions seen preoperatively (left panel) presented as largely healed necrotic lesions with renewed granulation 3 months after ECT (right panel)

*Patient 2* was an 83-year-old female with dementia at the time of ECT. She had previously undergone radio-chemotherapy in 12/2019 as part of the initial treatment upon diagnosis. Overall, the recurrence manifested as fairly extensive lesions in the region of the anterior commissure and also in the region of the introitus on the left side. The postoperative check-up 3 months after ECT revealed largely healed necrotic lesions with renewed granulation, as shown in Fig. 3.

**Fig. 4:**
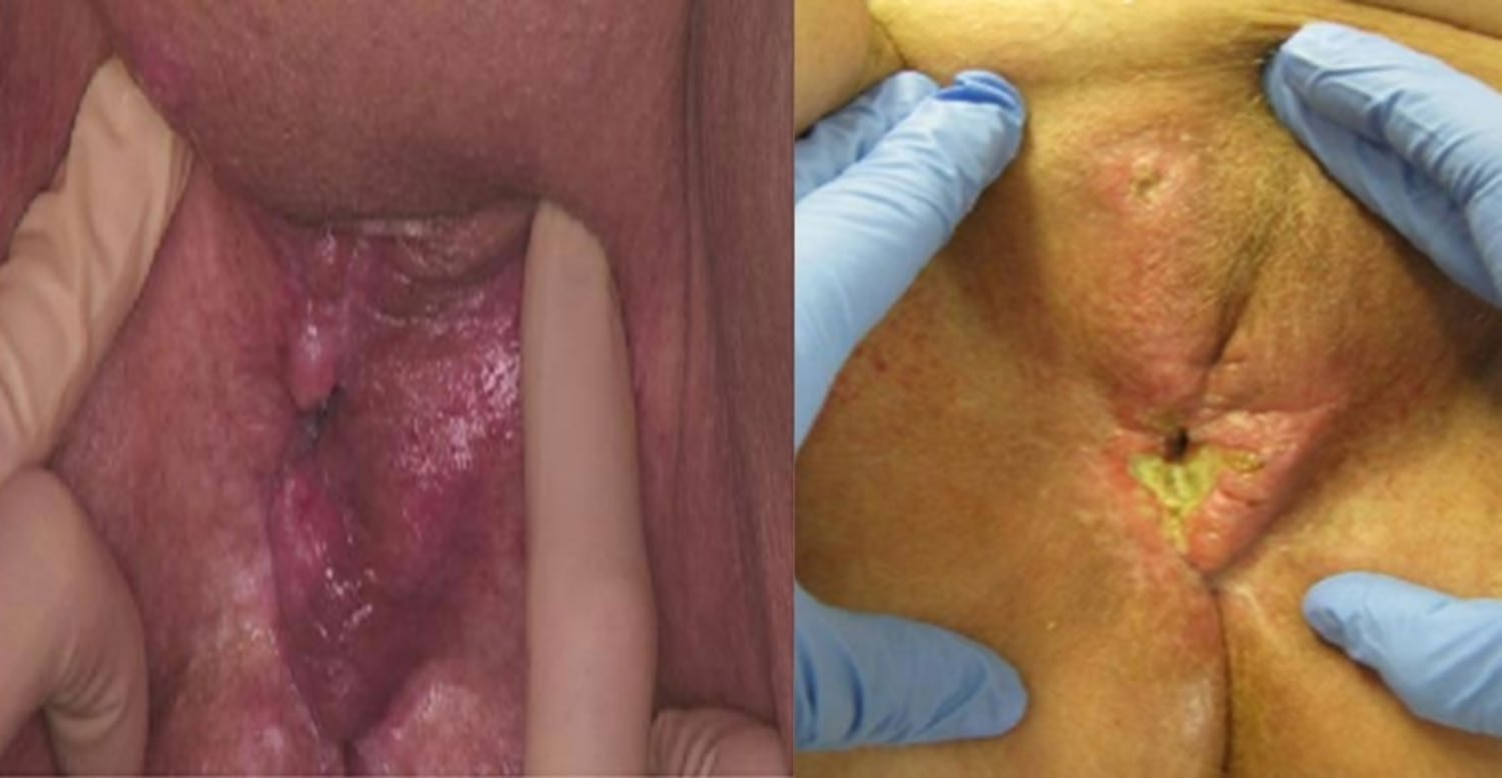
Foci of a third recurrence before and after electrochemotherapy (ECT). Following surgery for primary disease in 2011, the patient underwent surgery and radiation therapy for the first recurrence in 2014. The second recurrence in 2015 was treated surgically with plastic coverage. The foci of the third recurrence in 2016 were treated with ECT. The left panel shows the ECT operative site, while the right panel shows the lesions in the mons pubis region and vulvoperineal regions with necrosis in the process of healing 4 weeks postoperatively

*Patient 3* experienced primary disease in 2011 and underwent surgery. The first recurrence in 2014 was treated surgically, and the patient subsequently received radiation therapy. The second recurrence in 2015 was also treated surgically with plastic coverage. The foci of a third recurrence in 2016 were treated with ECT. Short-term monitoring at 4-week intervals showed lesions in the mons pubis and vulvoperineal regions with necrosis in the process of healing as illustrated by Fig. 4.

**Fig. 5:**
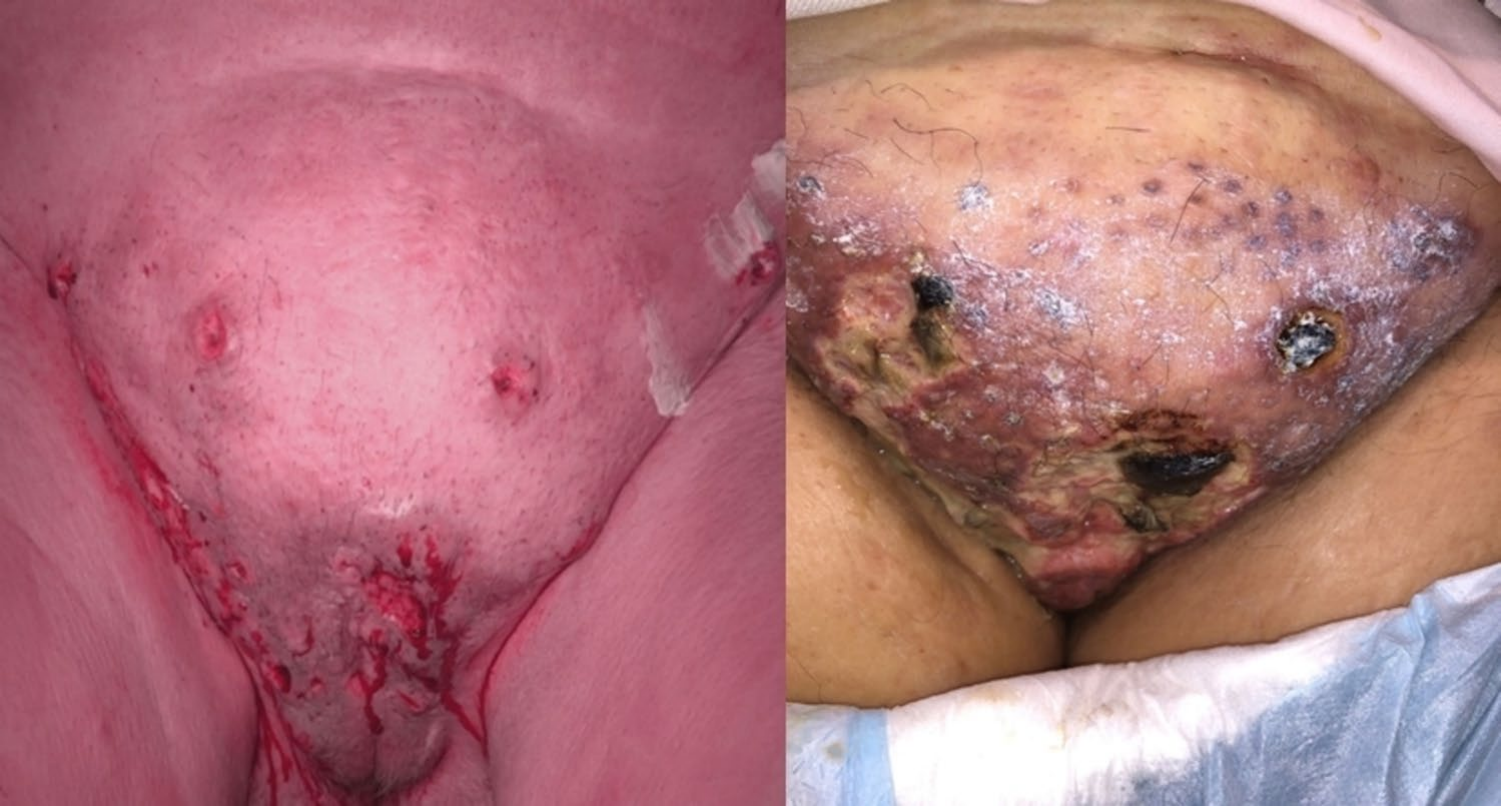
Mons pubis region of an 81-year-old patient after electrochemotherapy (ECT). After surgery and radiotherapy for the primary tumor in 2021 and surgical removal of the first recurrence in the mons pubis region in 2022, the second recurrence in 2023 showed extensive lesions in the mons pubis region, which extended into the region of the anterior vulva (left panel, image taken immediately before administering ECT). ECT appeared indicated due to multiple comorbidities. At 4 weeks post ECT (right panel), the lesions exhibited healing with necrosis of the larger foci and punctiform healing without further expansion of the foci, particularly in the mons pubis region

*Patient 4* underwent surgery in 2021 as part of the primary treatment, followed by radio-chemotherapy. In 2022, the first recurrence in the region of the mons pubis was removed by surgery. The second recurrence in 2023 showed extensive lesions in the mons pubis region, which extended into the region of the anterior vulva. Taking into account the patient’s numerous comorbidities, such as cardiomegaly, NYHA stage 3–4 heart failure, and atrial fibrillation, the decision was made to perform ECT. As shown in Fig. 5, the lesions exhibited healing with necrosis of the larger foci and punctiform healing without further expansion of the foci, particularly in the mons pubis region.

## Discussion

Conventional treatments such as radical resection in combination with radiotherapy, or radiotherapy alone, are generally not considered acceptable treatment options in patients, particularly elderly patients, with advanced vulvar carcinoma [12, 13]. In most cases, the numerous comorbidities that are often present cause problems when determining the best treatment options. In such cases, or in the recurrence setting after conventional therapy, ECT offers a treatment method that rapidly mitigates in the accompanying signs and symptoms and thus provides tumor control [14], especially as comparative studies of treatments for vulvar carcinoma are lacking. ECT also provides another advantageous effect in that it delays tumor progression and results in local destruction of tumor tissue. The indication for the use of ECT presented in the present study, i.e., locally advanced tumors or the desire or indication for palliative treatment, is consistent with published data [15]. Only one published study reports using ECT as a neoadjuvant treatment regimen [16].

In addition to age and comorbidities, recurrence is one of the main problems in the treatment of vulvar carcinoma. After primary treatment, more than 30% of patients with vulvar carcinoma experience recurrence [17–19], though many of these recurrences are in fact secondary tumors. The majority of all recurrences occur within the first two years after primary therapy [18–21]. In the present study, the recurrence-free interval ranged from 5 months to as much as 33 years, although in the latter case, the later tumor may have been a secondary tumor. According to the literature, vulvoperineal local recurrence is the most common location (in 53.4% of patients), followed by inguinofemoral recurrence (18.7%) and lymph node recurrence (up to 6%). Multiple recurrence sites are reported in as many as 14.2% of patients [6].

In general, there are no further treatment options for patients with recurrent or advanced local vulvar carcinoma after undergoing surgery, surgery and subsequent radiotherapy, or radiotherapy alone. In our study population, 12/15 patients had experienced at least one recurrence before undergoing ECT. Furthermore, while 8/12 patients experienced their first recurrence, the other patients had their second or even third recurrence, in one case even a fifth recurrence. Eight patients had undergone surgery followed by radiotherapy before receiving ECT treatment, while 4 patients had received radiotherapy alone. The latter group was particularly problematic in the recurrence setting. According to the guidelines [22] as well as in clinical practice, there are no other established therapeutic measures available that do not increase the burden of treatment on the patients. While systemic therapy should generally be avoided, such treatment was, nevertheless, necessary in 4/12 patients in the further course of the disease due to further progression or distant metastasis. In this respect, the interposed ECT afforded these patients an important gain in time until disease progression.

The disseminated occurrence of local recurrences is another significant factor in the recurrence setting. Lesions frequently extend beyond the vulva into the perineal region, as was observed in our study population, or may also involve the labia, if still present. In 5 of our patients, lesions also affected the mons pubis as an additional tumor location, most likely starting in the inguinal region, as shown in Fig. 5 as an example.

Due to the disseminated lesions, other treatment concepts based on local measures are out of the question. It should also be noted that local recurrence significantly worsens the patient’s prognosis. The shorter the disease-free interval, the less favorable the prognosis [2]. This fact must be taken into account particularly when deciding on further treatment measures. The primary goal must therefore be to improve the patient’s quality of life and to favorably influence or reduce the signs and symptoms that are associated with recurrence. Frequently reattempted local excisions carry the risk that defect coverage or tension-free defect coverage using myocutaneous flaps become impossible [21]. Pretreatment and tumor extent therefore significantly limit further treatment options [23].

In a comparable study by Corrado and collaborators [24], 15 patients with vulvar carcinoma were also treated using ECT. Their study reported on 14 patients with recurrences and one patient with primary vulvar carcinoma. In contrast to our study, 10 patients in their study had a single lesion, while the remaining 5 patients had disseminated tumor involvement.

In the follow-up setting after ECT, 5/7 patients in our study who received best supportive care were considerably advanced in age (83–92 years) at ECT. It is also noteworthy that periods of 6–8 months and more elapsed between diagnosis and the decision to administer ECT. Only in one case was therapy initiated promptly. This shows that no alternative therapy options were available or considered feasible or justifiable at the time of diagnosis. Two other patients had their first or second recurrence at the time of ECT. Both patients had previously received standard treatment in the form of surgery with radiotherapy or radiotherapy alone.

Radical surgery in the form of anterior and posterior exenteration was performed in two patients during the further course of the disease. In both these patients concerned, surgery was due to a third recurrence. Aged 69 and 74 at the time of ECT, both patients were markedly younger than the study population’s median age of 81 years at ECT. In both cases, radical surgery was preceded by surgical interventions and radiotherapy. The decision to perform exenteration was made 7 and 19 months after ECT, respectively. This meant that these patients gained a certain degree of progression-free survival in the treatment cascade.

Four patients were LTFU after ECT as they could not be contacted to ascertain their individual clinical status. Reasons for LTFU included known dementia-related cognitive impairment, age-related reduced general health, and comorbidities. This, in turn, confirms that ECT administration at the respective disease stages was the only justifiable treatment option.

The present study has its specific strengths and limitations. Our findings should therefore be regarded with some caution. Strengths of the study include that it adds to the hitherto sparse body of published data on the use of ECT in recurrent vulvar carcinoma. We found that ECT can provide symptom relief to patients in the palliative setting. The study has the usual limitations inherent in all open, nonrandomized clinical treatment studies that can recruit only small numbers of patients due to the relative rareness of cases. It is also unfortunate that 8 of our patients were 80 years or older at the time of ECT and unable to complete quality-of-life questionnaires and visual analog pain scales due to cognitive impairment or dementia.

## Conclusions

In agreement with the published literature, the present study also confirmed the value of ECT in the treatment of vulvar cancer in difficult settings. These patients are often elderly, have previously undergone multiple surgical and/or radiation treatments, and frequently have numerous comorbidities.

Therefore, the main criterion in our study was the improvement of patients’ quality of life in terms of pain relief and the reduction of clinical symptoms, such as bleeding, itching, odor, and secretion. To this end, this study specifically recorded parameters, such as pain and local tumor control, in terms of reducing concomitant clinical signs, specifically bleeding due to tumor-related ulceration, and secretion.

ECT was performed using bleomycin under laryngeal mask anesthesia to provide adequate analgesia. Taking potential side effects into account and using appropriate supportive care, the treatment was well tolerated by all patients.

In our observation, the treatment goals of ECT were achieved. Subsequently, the further course of the disease and, in particular, any necessary further interventions remain to be of interest.

## Data Availability

No datasets were generated or analysed during the current study.
